# Association of zygotic piRNAs derived from paternal *P* elements with hybrid dysgenesis in *Drosophila melanogaster*

**DOI:** 10.1186/s13100-018-0110-y

**Published:** 2018-02-06

**Authors:** Keiko Tsuji Wakisaka, Kenji Ichiyanagi, Seiko Ohno, Masanobu Itoh

**Affiliations:** 1 Department of Applied Biology, Kyoto Institute of Technology, Hashigamicyo Matsugasaki, Sakyo-ku, Kyoto, 606-8585 Japan; 2 Laboratory of Genome and Epigenome Dynamics, Department of Applied Molecular Biosciences, Graduate School of Bioagricultural Sciences, Nagoya University, Nagoya, 464-8601 Japan; 3 Center for Epidemiologic Research in Asia, Shiga Univesity of Medical Science, Otsu, Shiga 520-2192 Japan; 4 Center for Advanced Insect Research Promotion (CAIRP), Kyoto Institute of Technology, Kyoto, 606-8585 Japan

**Keywords:** Zygotic piRNAs, Paternal *P* elements, piRNA cluster, Hybrid dysgenesis, Natural strains

## Abstract

**Background:**

*P*-element transposition in the genome causes P-M hybrid dysgenesis in *Drosophila melanogaster*. Maternally deposited piRNAs suppress *P*-element transposition in the progeny, linking them to P-M phenotypes; however, the role of zygotic piRNAs derived from paternal *P* elements is poorly understood.

**Results:**

To elucidate the molecular basis of *P*-element suppression by zygotic factors, we investigated the genomic constitution and *P*-element piRNA production derived from fathers. As a result, we characterized males of naturally derived Q, M’ and P strains, which show different capacities for the *P*-element mobilizations introduced after hybridizations with M-strain females. The amounts of piRNAs produced in ovaries of F1 hybrids varied among the strains and were influenced by the characteristics of the piRNA clusters that harbored the *P* elements. Importantly, while both the Q- and M’-strain fathers restrict the *P*-element mobilization in ovaries of their daughters, the Q-strain fathers supported the production of the highest piRNA expression in the ovaries of their daughters, and the M’ strain carries *KP* elements in transcriptionally active regions directing the highest expression of *KP* elements in their daughters. Interestingly, the zygotic *P*-element piRNAs, but not the *KP* element mRNA, contributed to the variations in *P* transposition immunity in the granddaughters.

**Conclusions:**

The piRNA-cluster-embedded *P* elements and the transcriptionally active *KP* elements from the paternal genome are both important suppressors of *P* element activities that are co-inherited by the progeny. Expression levels of the *P*-element piRNA and *KP*-element mRNA vary among F1 progeny due to the constitution of the paternal genome, and are involved in phenotypic variation in the subsequent generation.

**Electronic supplementary material:**

The online version of this article (10.1186/s13100-018-0110-y) contains supplementary material, which is available to authorized users.

## Background

Transposable elements (TEs) are major structural constituents of eukaryotic genomes. Although their mobilization provides genetic variation and drives genome evolution [1, 2], TEs exert deleterious effects on the host. For example, TE mobility in *Drosophila melanogaster* causes germline abnormalities known as hybrid dysgenesis (see below for details). The host counteracts this deleterious effect through various pathways, including Piwi-interacting small RNAs (piRNAs).

piRNAs are small non-coding RNAs that are generally 24–35 nucleotides (nt) long and act to suppress TE expression [3]. piRNAs are generated from particular genomic loci, called piRNA clusters that consist of many TEs. Two types of piRNA clusters have been identified in *D. melanogaster*; dual-strand and unistrand clusters are dominant in germline cells and somatic cells, respectively. In the dual-strand piRNA cluster, transcription occurs in both directions to produce long precursor single-stranded RNAs that are subsequently chopped into 24- to 35-nt RNAs. These are loaded onto Piwi-family proteins to direct the cleavage of complementary RNAs, including TE mRNAs. The cleaved RNAs are then loaded onto a Piwi family protein to aid in the cleavage of complementary based RNA, a reaction known as the “ping-pong cycle.” In the unistrand piRNA cluster, long precursor RNAs are transcribed in a single direction. Because TEs are inserted predominantly into the unistrand piRNA clusters in the reverse orientation to the precursor transcription, they can serve as a source of TE-derived antisense piRNAs that are used by the PIWI protein to induce repressive chromatin modification [4–9]. Owing to these biogenesis pathways, piRNAs are generated and retained primarily in the cytosol, although a fraction of them are transported into the nucleus. One hundred forty-two piRNA clusters have been identified in the *D. melanogaster* genome, but piRNA production levels supplied by these clusters are highly variable [3]. Clusters with high piRNA production are called active piRNA clusters, while others are referred to here as low activity piRNA clusters.

*P* elements are DNA transposons that propagate in the *D. melanogaster* genome and include both structurally complete and incomplete variants. Autonomous 2907-bp complete elements encode an 87-kDa transposase, for which expression can be detected in germline cells [10–12]. *P* elements are responsible for a phenomenon called “P-M hybrid dysgenesis.” Progeny of a cross between an M-strain female with no *P-*element and a P-strain male carrying complete *P* elements demonstrate increased frequencies of *P*-element transposition resulting in germline cell abnormalities. These abnormalities can include gonadal dysgenesis (GD) with sterility, chromosomal breaks, mutations, and male recombination [13–16]. Therefore, although recent reports argue against the involvement of the *P* transposition in GD [17, 18], previous reports indicate that P-strain males have a high ability to mobilize *P* elements in their progeny (high *P* inducibility), and M-strain females are not able to repress *P* transposition (high *P* susceptibility) [19–24].

When P-strain males are mated with P-strain females, *P*-element mobilization in the germline cells of their progeny is prevented by maternally deposited repressors [14, 25]; therefore, P-strain females have low *P* susceptibility. It has been proposed that the GD phenotype in female progeny (i.e., *P* susceptibility) is determined largely by cytoplasmic factor(s) in the maternal oocytes, rather than by the genotype of either the daughters or the mothers. Thus, the oocytes are distinguished as “cytotypes.” M-strains females produce oocytes of “M cytotype,” which produce dysgenic daughters when crossed with a P-strain male. P strain females produce oocytes of “P cytotype,” which produce normal daughters. The major molecular entity that determines the P-M cytotype in oocytes has been proposed as a cytosolic *P*-element piRNA that is inherited by the daughters to suppress *P* transposition [26, 27]. It also has been reported that *P* mobilization in progeny is controlled by other factors, such as proteins produced from full-length (type I, 66-kDa repressors) and internally deleted elements (type II, *KP* repressors) [25, 28–34]. The *KP* elements, non-autonomous incomplete variants with a nucleotide deletions at 808–2060, are present ubiquitously in natural populations [28, 29, 35] and supply the most common type II repressor protein that inhibits *P*-element transposition [29–33, 36, 37].

The Q and M′ strains have distinct characteristics from P and M strains and are of great interest. The M′ strains carry *P*-element copies or *P*-element-like copies in their genomes, but they behave as M strains. Thus, when M′-strain females are crossed with P-strain males, *P*-elements are transposed. The Q strains also carry P elements that are not mobilized, even upon paternal transmission. The difference between M′ and Q is the P susceptibility; when Q-strain females are crossed with P-strain males, *P* transposition is prevented. In a previous study, we proved that the M′-strain progeny produced lower levels of maternal piRNAs than the Q-strain progeny [38]. On the other hand, when M-strain females are crossed with Q- or M′-strain males (Q or M′ hybrids), *P* transposition is prevented, although the mechanisms are not fully elucidated. Thus, the Q and M′ strains have low *P* inducibility, despite the presence of *P* elements in their genomes. The Q and M′ strains are most common in the natural populations in Eurasia, Africa, Australia, and the Far East [18, 39, 40]. It has been reported that *KP* and *SR* polypeptides, produced from non-autonomous incomplete *KP* and *SR* elements, respectively, and found on the paternally inherited chromosomes, play an important role in regulating *P* transposition [36, 41]. The positional effects also are involved in regulating *P* inducibility [42, 43]; however, it is unknown whether *P*-element piRNAs produced from the paternally inherited chromosomes (zygotic piRNAs) play a role in the regulation of *P* transposition in the progeny. In particular, it is largely unclear how zygotic piRNAs are produced in Q and M′ hybrids, and whether they influence the P-M phenotypes. Furthermore, although the abilities of the F2 hybrids to suppress *P* transposition are considerably varied [44, 45], it is unknown how a male genome contributes to the immunity of the produced granddaughters.

In the present study, we used four fly lines from wild-sampled Q, M′, and P strains as paternal lines, and then analyzed the following points to elucidate the paternal effects on the P-M phenotype: (1) the effects of the paternally inherited genome on the cytotype of F1 oocytes, (2) the fraction of each *P*-element type (e.g., *FP*, *KP*, and non-*KP*) present in the respective genomes, (3) the expression levels of *P* and *KP* elements, (4) the genomic positions of their insertions and the transcriptional activity of these insertion sites, (5) the number of *P* elements embedded in each piRNA cluster, and (6) the amount of piRNA production in whole embryos and ovaries of the F1 progeny, obtained by crossing with an M-strain female. As a result, we revealed that the paternally inherited Q and M′ genomes can serve as sources of zygotic piRNAs in the progeny, even at young ages; the amounts vary depending on the *P* elements embedded in the piRNA clusters. These zygotic piRNAs acted to reduce the amount of *P*-element mRNA. Furthermore, these piRNAs affected the P-M phenotypes of the F2 progenies. Thus, upon paternal inheritance, the Q and M′ genomes can co-transmit these *P-*element piRNA–generating immunity loci with complete *P* elements. In addition, high ratios of *KP* element transcription in the Q and M′ genomes likely are associated with the repressive transcriptional states of genomic regions surrounding *P* elements and appear to play a regulatory role. Moreover, the Q-strain males conferred immunity against *P*-element transposition to their granddaughters, which not only underscores the important role of piRNA cluster-inserted *P* elements in the regulation of *P*-element transposition, but also offers a genetic basis for the prevalence of Q-type flies in natural populations.

## Methods

### Fly stocks

The following nine isofemale *D. melanogaster* lines were used: M′-OM5 [43] as the M′ strain; Q-KY74 (KY-02-074) and Q-KY101 (KY-02-101) as the Q strains, and Q-HKH (Hikone-H 1957) [46] as the Q strains in part. Harwich (P-Har) males and Canton S (M-CS) females were used as standard P and M strains, respectively.

### Gonadal dysgenesis test

GD tests were used to determine *P* inducibility and *P* susceptibility in the P-M system [14, 47] Two types of crosses were performed as follows: cross A (M-CS females x tested males) and cross A* (tested females x P-Har males). One- to four-day-old hybrid females of each line were dissected at same time. By analyzing approximately 50 F1 or 100 F2 hybrid females from each line, the GD score was calculated as the percentage of females having dysgenic ovaries. For the analysis of F1 hybrid GD scores, test males were crossed with M-CS and maintained at 28 °C where GD becomes obvious. For the analysis of F2 hybrid GD scores, F1 hybrid were maintained at 25 °C because they are fertile at this temperature, then the F1 hybrid females were crossed with P-Har and the F2 progeny was incubated at 28 °C.*P* inducibility was determined by GD scores in cross A. The criteria for low *P* inducibility was GD < 10.0%. *P* susceptibility was determined in cross A*. The criteria for low *P* susceptibility was GD < 10.0% in cross A* [19].

### PCR and quantitative PCR

Genomic DNA was extracted from whole bodies of 20–40 flies from each line with standard methods [48]. These DNAs were used for polymerase chain reaction (PCR) as a template with two sets of primers: one to amplify total *P* elements and the other to amplify non-*KP* elements. The PCR products then were sequenced. Quantitative amplification of DNA was performed, using primer pairs specific to the *KP* element and total *P* element, respectively. The single-copy *RP49* gene was used for normalization [49]. Details are shown in the Additional file 1.

### Deep sequencing of the *P*-element insertion site

The genomic insertion sites for *P* elements were amplified according to the protocol of Tsukiyama et al. [50] with minor modifications. The genomic DNA extracted from 40 adult flies was digested with *Hha*I or *Taq*I (TaKaRa, Japan) and ligated to overhanging adapters. Using these ligation products as a template, PCR was performed with primers specific to the adaptor and to *P* elements respectively. Nested PCR was performed to specifically amplify P-element-containing PCR fragments. About 300- to 600-bp-long *Hha*I and *Taq*I products were purified from an agarose gel, and used for preparation of deep sequencing libraries with the TruSeq DNA PCR-Free LT Library Prep Kit (Illumina, California, USA). Pair-end 250-bp sequencing was performed on the MiSeq system (Illumina). Details are shown in the Additional file 1.

### Analysis of insertion site

The obtained deep-sequencing data was analyzed, as previously described [27], using the CLC Genomics Workbench (QIAGEN Bioinformatics, Denmark; detailed protocol: https://www.qiagenbioinformatics.com/support/manuals/) with minor modifications. Reads with no *P*-element sequence were discarded. Adaptor sequences were removed by the “transcriptome analysis” function in g_x_and then the sequences were mapped to the *D. melanogaster* genome (Release 5) using the “download genome” function in g_x_ to identify insertion sites for *P* elements. To normalize the occupancyof each insertion site in the population, the number of reads supporting respective insertion sites was divided by the total reads. The transcriptional states of the identified genomic sites were analyzed with *D. melanogaster* Genome Browser in ModENCODE (Generic Genome Browser, v. 2.52; GMOD), and the read numbers of *P* elements inserted into piRNA clusters were analyzed according to Brennecke et al. [3].

### Analysis of piRNA clusters

The piRNA clusters were divided into two groups (unistrand and dual-strand) according to percentages of piRNA strand distribution, as reported by Brennecke et al. [3]. The 142 genomic locations are shown as sites of abundant piRNA generation in *Drosophila* ovaries. If both sense and antisense strands in the piRNA cluster are > 20.0%, we considered the piRNA cluster to be “dual-stranded,” with the ability to produce both strands of piRNA. Others were considered unistrand. The active piRNA clusters are the top 15 clusters ranked by the number of cluster-unique piRNAs [3].

### RNA preparation

Total RNA was extracted from 0-h to 24-h embryos from forty cross A couples with the miRNeasy Kit (QIAGEN), and small RNAs were separated using the RNeasy MinElute Cleanup Kit (QIAGEN). F1 females from 20 couples were grown at the GD-inducing temperature of 28 °C for 4–7 days [14, 47], and then total RNA was extracted from the approximately 8 normal ovaries of those female progeny at 2- to 3-days old. The dysgenic ovaries of P-Har hybrids were dissected from approximately 100 females. The testes were dissected from 60 males of each line, and total RNA was extracted from the pooled testes.

### Small RNA sequencing

Small RNA libraries were prepared using 1 μg of small RNAs with the TruSeq Small RNA Sample Preparation Kit (Illumina). After PCR amplification, products of approximately 150 bp were extracted from a 6% polyacrylamide gel. Single-end 50-bp sequencing of these libraries was performed using the MiSeq system (Illumina). The obtained small RNA reads were analyzed and annotated as described previously [38].

### RT-PCR and quantitative RT-PCR

cDNAs were synthesized by superscript III reverse transcriptase (Invitrogen) using total RNA and an oligo-dT primer. Quantification of cDNAs was performed by real-time PCR using primer pairs specific to the *KP* element and total *P* element, respectively. Details are shown in Additional file 1.

#### Statistical analyses

Pearson product–moment correlation tests were conducted using R (ver. 3.0.2).ierarchical cluster analyses were conducted using R and Excel with the hclust function (the furthest-neighbor method). Student ***t*** tests were conducted using Excel.

#### Data availability

Sequence data are available at DDBJ under the accession number, PRJDB5877.

## Results

### The effects of the paternal genome on the P-M system

**Fig. 1:**
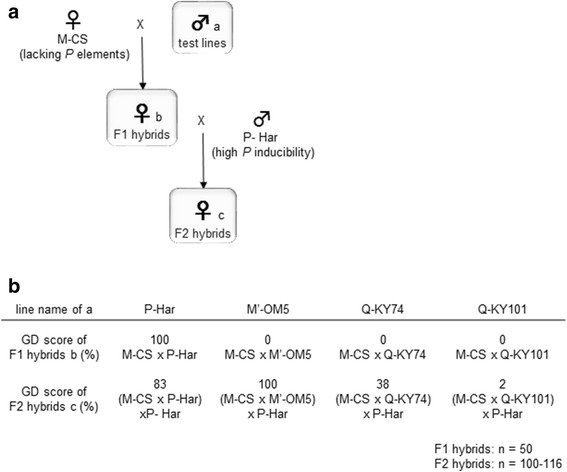
GD phenotypes of the F1 and F2 hybrids from the P-, M′- and Q- strain males (**a**) Schematic representation of the experimental design for crosses. The test males (**a**) of the four line were crossed with the M-CS females to obtain F1 females (**b**), which were then crossed with P-Har males to obtain F2 females (**c**). **b** GD scores of F1 and F2 hybrids. Ovaries of approximately 50 (F1) and 100 (F2) flies were investigated to score the GD

To study the effects of the paternally inherited genome on the mobility of *P* elements in progeny, fly lines of Q (Q-KY74 and Q-KY101), M′ (M′-OM5), and P strains (P-Har) were analyzed. When males from these lines (i.e., fathers; test strain) were crossed with females from the *P*-element-susceptible M-CS strain (i.e., mothers), the F1 progeny showed 100% GD for P-Har males, and 0% GD for the others (Fig. 1a, b), as previously reported [38, 43], confirming that P-Har males have high *P* inducibility; Q-KY74, Q-KY101, and M′-OM5 males had no *P* inducibility, despite carrying *P* elements. These results suggest that some repressive factors are co-inherited with *P* elements from M′ and Q fathers, and then expressed in F1 ovaries.

It is possible that these repressive factors also affect the cytotype of oocytes of F1 hybrids. To examine this, we performed GD tests for F2 hybrids from a cross between F1 hybrid females and P-Har males. If the paternally inherited genome served as a source of cytoplasmic repressive factors in the F1 oocytes, the F2 hybrids should have shown resistance against *P* elements (i.e., low GD score). Interestingly, F2 hybrids [(M-CS x test males) females x P-Har males] showed considerable variability in GD scores (Fig. 1b). When M′-OM5 was used as a test strain, F2 hybrid offspring showed a GD score of 100%. Thus, although the M′-OM5 genome inhibited repressive factors of *P* transposition in F1 ovaries (see above), it did not alter the cytotype of F1 oocytes. In contrast, when Q-KY101 was used as a test strain, F2 female offspring showed a very low GD score (2%), suggesting that the Q-KY101 genome conferred the *P*-resistant cytotype to the F1 oocytes. The Q-KY74 F2 hybrid offspring also demonstrated a low GD score (38%), although not as low as Q-KY101 F2 hybrids. When P-Har males were used as test males, the F2 progeny showed a high GD score, as seen previously [45]. It should be noted that the F1 hybrids resulting from crosses between M-CS females and P-Har males were fertile when grown at 25 °C, and that GD tests were conducted at 28 °C to enhance dysgenic effects.

To determine the paternally inherited factors that contributed to the suppression of *P* transposition in F1 and F2 hybrid offspring, we examined the expression levels of *P*-element piRNAs and those of *KP*-element mRNAs, both of which are known to be main repressors in germ line cells.

### The Q and M′ strains possessed high ratios of *KP* elements and low fractions of *FP* elements

**Fig. 2:**
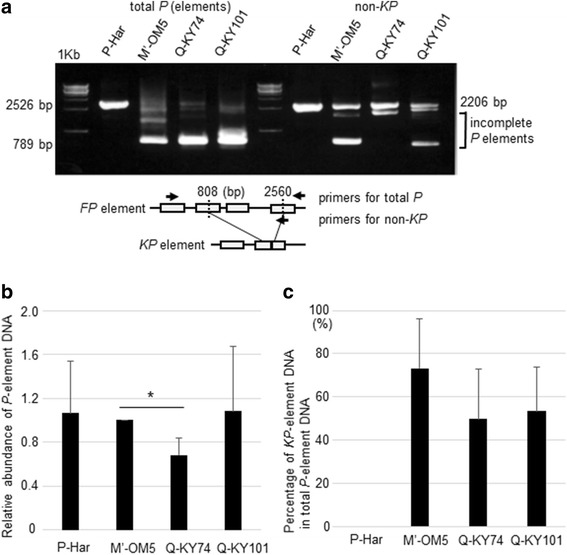
Genomic composition of *P* elements (**a**) Top: Results of PCR analysis amplifying total *P* elements (left, labeled with total *P* on the top) and non-*KP* elements (right, labeled with non-*KP*) using the respective genomic DNAs indicated on the top of each lane. The positions of DNA bands derived from *FP* (2526 bp) and *KP* (789 bp) elements produced in the “total *P*” PCR are indicated on the left. The positions of bands derived from the *FP* (2206 bp) and incomplete (internally deleted) elements produced in the “non-*KP*” PCR are indicated on the right. Bottom: Structure of the FP and KP elements and the primer design used for the PCR. The deletion junctions (at 808 and 2560 bp) of the KP element are indicated. **b** The copy numbers of *P* elements in the respective genomes relative to that in the M′-OM5 genome. Star (*) indicates statistical significance (*p* < 0.05). **c** The ratio of *KP* elements to the total *P*-element copies quantified by qPCR with KP- and total-P-specific primers

First, we characterized the *P*-element copies in the fly lines Q-KY74, Q-KY101, M′-OM5, and P-Har by PCR using two sets of primers designed for total *P* and non-*KP* elements, respectively (see Fig. 2a for primer design). The “total *P*” primers allowed for amplification of both *FP* elements and incomplete (internally deleted) *P* elements, but if incomplete elements were predominant in the genome, the *FP* element amplicon (2526 bp) would not be produced efficiently. The “non-*KP* primers” allowed for amplification of *FP* elements, even in the presence of a large number of *KP* elements (e.g., Q-KY74, Q-KY101, and M′-OM5). Using the total-*P* primers, we detected an amplicon (2526 bp, sequence confirmed) of *FP* elements from P-Har genomic DNA (Fig. 2a). DNA from strains Q-KY74, Q-KY101, and M′-OM5 revealed a faint *FP*-element band and a thick *KP*-element band (789 bp). The presence of *FP* elements in the Q-KY74, Q-KY101, and M′-OM5 genomes were confirmed by PCR using the non-*KP* primers (Fig. 2a); these genomic DNAs revealed an *FP* amplicon (2206 bp), along with amplicons from incomplete variants. These results indicate that Q-KY74, Q-KY101, and M′-OM5 indeed carry *FP* elements, although the vast majority of their genomic *P* elements are *KP* elements.

To determine relative copy numbers of *P* elements in the four genomes, we performed real-time PCR with primers that amplified *P* copies (Fig. 2b and Additional file 2: Figure S1; three biological replicates for each line). Results revealed that these strains contained similar numbers of *P*-element copies, although the copy number in the Q-KY74 genome was somewhat (0.7-fold) lower than those in the other genomes. It should be noted, however, that standard deviations were large, indicating that *P*-element copy numbers varied significantly among individual flies of the same line. To determine relative copy numbers of *KP* elements, we performed real-time PCR with *KP*-specific primers (Fig. 2c; three biological replicates for each line). The Q-KY74, Q-KY101, and M′-OM5 genomic DNAs amplified *KP* elements, while the P-Har genomic DNA did not. Although copy numbers varied between individuals of the same lines, as was the case with *P* elements, the M′-OM5 genome showed a higher ratio of *KP* copy numbers (73% of total *P* elements) than the two Q strains (~ 50% of total *P* elements), which is consistent with our previous results from M′-OM5 using Southern blotting [43].

These results suggest that the number of *P* elements in the genome is not attributable to the differences in GD scores from F2 hybrids among the P, M′ and Q strains. However, regarding F1 hybrids, the strains that showed low GD scores (M′ and Q) possessed high ratios of *KP* elements in their genomes. This suggests the contribution of paternally inherited *KP* elements in the suppression of the *P*-element expression and/or transposition in F1 hybrids. As a result, we chose to investigate the expression of *P* elements, including *KP* elements, as shown below.

### The Q strains possessed high percentages of *P* elements inserted into repressive regions

To investigate whether chromosomal environments of *P*-element insertion sites in the paternal genome influence the level of *P*-element expression in F1 hybrid ovaries, we first determined the *P*-element insertion sites in the respective genomes. We then inferred the transcriptional states of the surrounding regions in ovaries of a *D. melanogaster* reference line. To identify the insertion sites, we digested genomic DNA (pooled for 40 adults) with restriction enzymes, and ligated an adaptor DNA to the ends. Junction regions between *P* elements and their flanking sequences were amplified using a *P*-specific primer, an adaptor-specific primer, and the ligated DNA. The PCR products were then subjected to paired-end deep sequencing and mapped onto the reference *D. melanogaster* genome (Release 5). This identified a number of insertion sites in the respective genomes (Fig. 4a). We noticed that the number of sequencing reads significantly differed among loci. This variability most likely stemmed from differences in occupancies of the respective sites. For insertion sites with low coverage, it is conceivable that only a fraction of the individuals carried the insertions (i.e., insertional polymorphism among individuals of the same line).

**Fig. 3:**
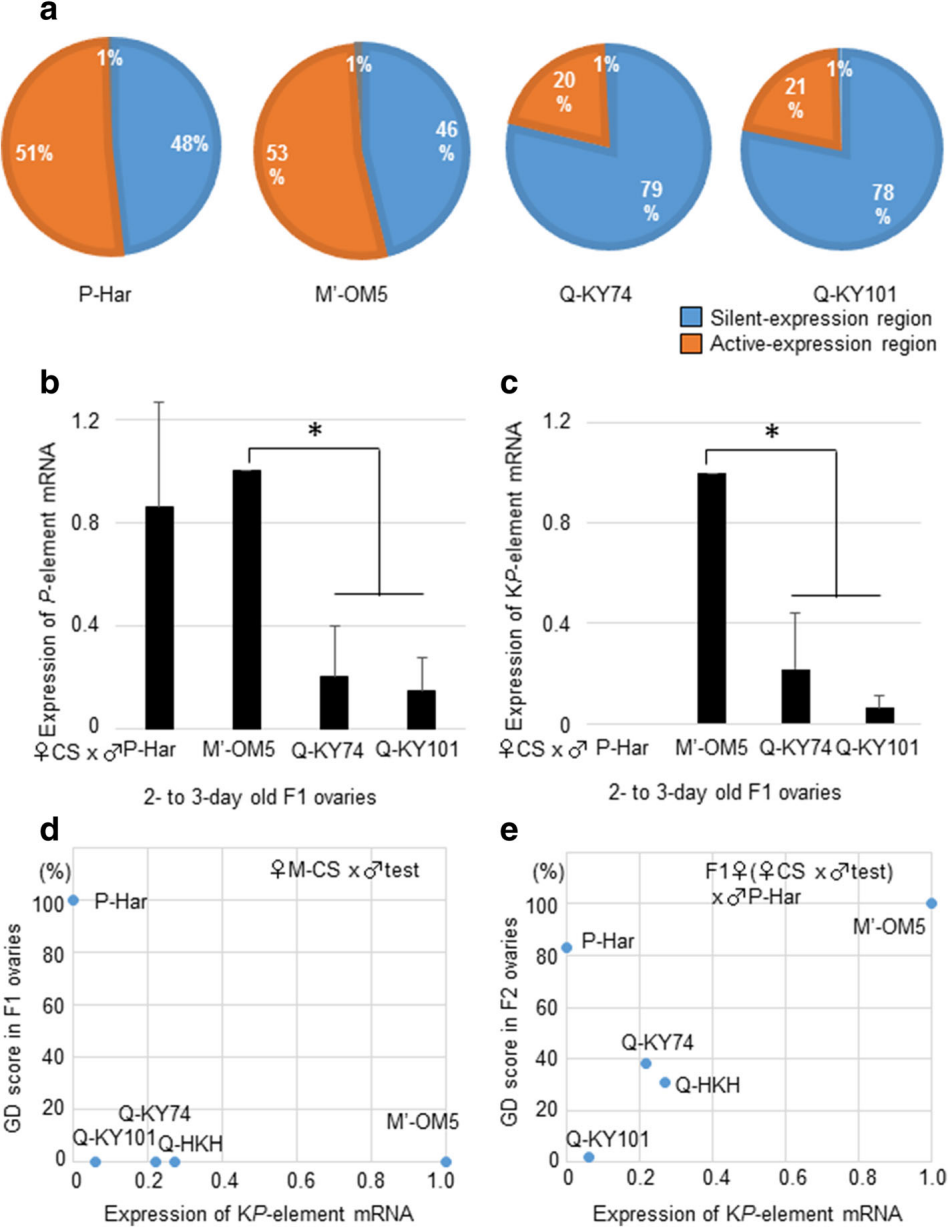
Expression of *P*-element and *KP*-element mRNAs in ovaries and their relationships to GD scores (**a**) Fractions of silent- and active-expression regions that harbor *P* elements in the respective genomes. The fractions were calculated by dividing the number of sequencing reads that supported insertion of the respective regions by the total sequencing reads to represent occupancy-adjusted copy numbers. 1% represents the fraction of unknown region that harbors *P* elements in the respective genomes. **b**, **c** The levels of *P*-element mRNA (**b**) and *KP*-element mRNA (**c**) in young F1 ovaries. The expressions were quantified by qRT-PCR and normalized by those of M′-OM5 hybrids using qRT-PCR. Star (*) indicates statistical significance (*p* < 0.05). **d**, **e** The relationship between the level of *KP*-element mRNAs in F1 ovaries (x-axis) and GD scores (y-axis) of F1 (**d**) and F2 (**e**) progenies

Next, the transcriptional states of the flanking regions were categorized into active-expression regions (score > 0) and silent-expression regions (score = 0) (Fig. 3a), according to the RNA-seq data from 4-day-old ovaries in *D. melanogaster* Genome Browser (ModENCODE) [51, 52]. Using these data, the numbers of *P*-element copies inserted in the respective transcriptional states were calculated. We determined the fraction of read numbers (rather than number of insertion sites) mapped in the two regions so that the occupancy of respective insertion sites was taken into account (Fig. 3a). In the P-Har genome, about half of the *P* elements were inserted into active-expression regions. Because nearly all *P* elements are *FP* elements in P-Har (Fig. 1a), it is likely that the *FP* copies inserted in the active-expression regions were expressed in F1 overies, resulting in high *P* inducibility. On the other hand, in the Q-KY74 and Q-KY101 genomes, more than three-quarters of the *P* elements (including *FP* and *KP*) resided in silent-expression regions, consistent with showing no *P* inducibility (Fig. 1b, F1 GD scores); however, this could not completely explain the variable GD scores for both F1 and F2 hybrids. In M′-OM5, about half of the *P* elements resided in active-expression regions, although they were not dysgenic.

### The M′ strain expressed higher levels of *KP* elements, while the Q strain transcribed lower levels of *P* elements

Next, we quantified *P*-element mRNA in 2- to 3-day-old ovaries of F1 hybrids, using real-time PCR. As expected from the features of *P* insertion sites, P-Har hybrids showed a high expression of the *P* element (Fig. 3b). *P*-element expression in M’-OM5 hybrids was also high, and notably, it was 5-fold and 10-fold higher than expression in the Q-KY74 and Q-KY101 hybrids, respectively (*p* < 0.01; Fig. 3b). Given that the number of *P*-element copies inserted in transcriptionally active regions differed only by 2.5-fold between the M′ and Q strains, some factors other than genomic loci were likely responsible for the very low expression levels of *P* elements in the hybrid offspring from Q-KY74 and Q-KY101 strains.

We determined the levels of *KP*-element mRNA in the same samples (2- to 3-day-old ovaries of F1 hybrids) using *KP*-specific primers (Fig. 3c). While P-Har hybrids did not express detectable *KP* mRNA, M′-OM5, Q-KY74, and Q-KY101 hybrids did show *KP* expression; M′-OM5 hybrids expressed higher levels of *KP*-element mRNA than both Q-KY74 and Q-KY101 (p < 0.01; Fig. 3c). Although the abovementioned genomic site identification (shown in Fig. 3a) did not discriminate between *FP* and *KP*, a substantial number of *KP* elements should have been in active-expression regions in the M′-OM5 genome; therefore, higher levels of *KP* expression in M′-OM5 are consistent with the abundant insertions in active-expression regions in this strain of flies.

As mentioned above, we noticed that the M′-OM5 hybrids expressed a substantial amount of *P*-element mRNA, although they were not dysgenic. The non-dysgenic phenotype could be due to the concomitant expression of the *KP* repressor mRNA in the ovaries. Using the data from the four strains and an additional Q strain, Q-HKH, a simple comparison of *KP*-element mRNA expression and GD scores from the F1 hybrids for the respective strains did not indicate a strong correlation (Fig. 3d) like that seen in F2 hybrids (Fig. 3e). However, we noted that F1 hybrids of the Q strains expressed *P*-element piRNAs, another repressor molecule (see below); therefore, it remains possible that the *KP* mRNA plays an important role in the prevention of GD in M′ F1 hybrids. We will discuss this possibility later.

Thus, the relative abundance of *P*-element insertions in active-expression regions is roughly correlated to the level of *P*-element mRNA expression, but it did not fully account for the difference in expressions between the M′ and Q strains. Thus, other factor(s) also should be involved in the control of the *P*-element mRNA levels and the P-M phenotype. Such factors may involve piRNAs.

### The Q strains had more copies of *P*-element in piRNA clusters

piRNAs are produced from the transcripts of piRNA clusters [3]. To reveal whether the level of *P*-element piRNAs in F1 hybrid ovaries was affected by the number of *P*-element copies inserted into the clusters in paternal genomes, we first re-analyzed the insertion site data of the four lines in view of the number of *P*-element reads identified in the piRNA clusters. The characteristics of piRNA clusters (genomic locations, piRNA-transcriptional directions, and piRNA-producing activities) were compared to the data presented in [3]; (see “Methods”). Here, active piRNA clusters have been defined as the top 15 clusters, ranked by the number of unique piRNAs they provide, whereas the remaining clusters have been defined as low activity piRNA clusters.

**Fig. 4:**
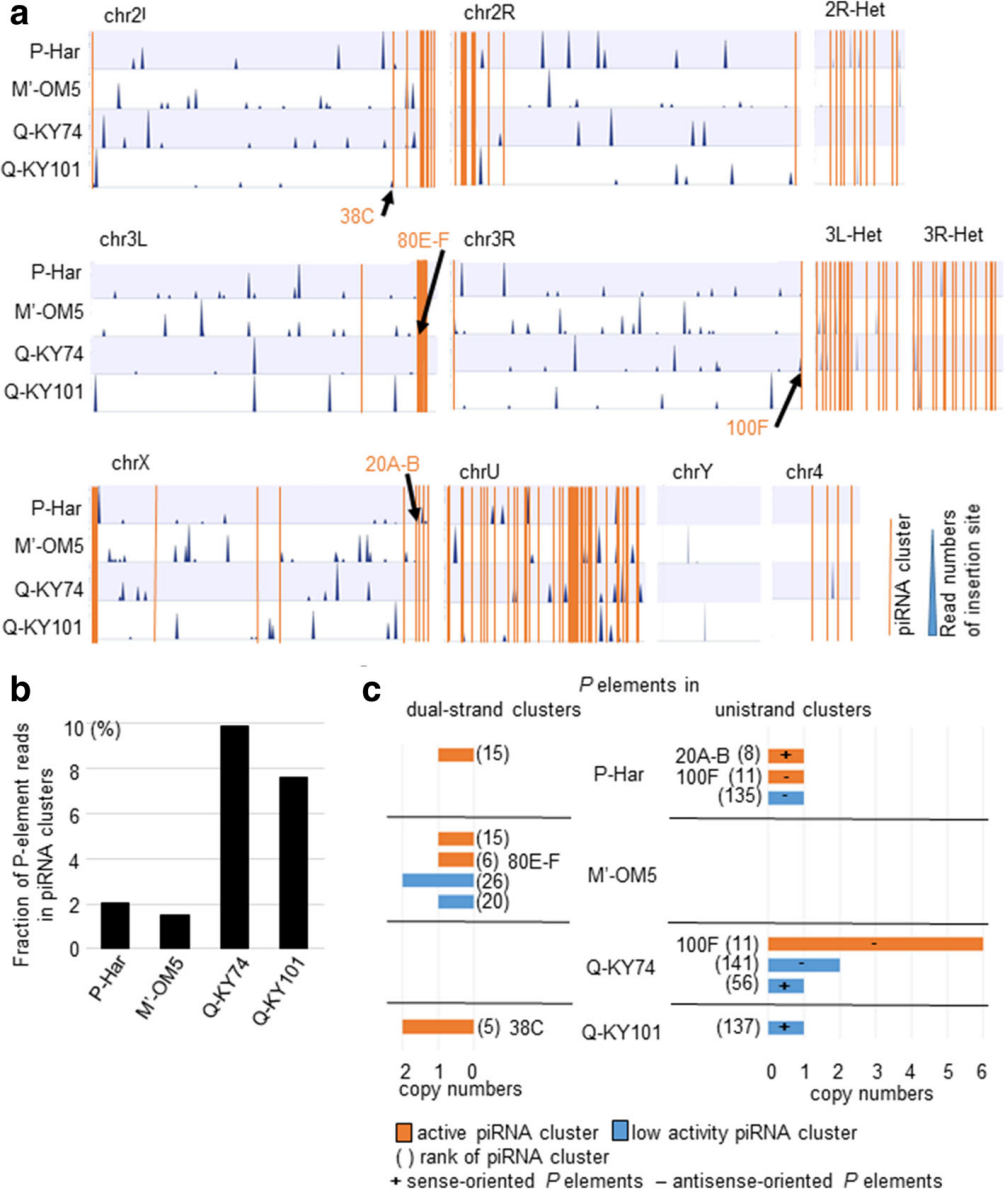
Identification of *P*-element copies inserted into piRNA clusters (**a**) Genomic distributions of *P*-element insertion sites identified in the respective genomes (blue triangles). The height of blue triangles represents relative numbers of sequencing reads that supported insertion. Orange blocks show the piRNA [3]. Arrows indicate the active piRNA clusters harboring *P* elements. **b** The percentages of *P*-element reads identified in piRNA clusters in the total *P*-element reads. **c** Number of *P*-element copies in dual-strand (left) or unistrand (right) piRNA clusters. Active and low activity piRNA clusters are shown in orange and blue, respectively. Cluster names are shown with their rank of expression ability. The. + and - show the sense and antisense *P*-element insertion in relation to the transcription of the unistrand cluster

In all lines analyzed, some *P*-element copies were located in piRNA clusters (Fig. 4a), but not all lines harbored *P* elements in the same clusters; only Q-KY74 and P-Har had *P*-element insertions in the same100F piRNA cluster. The fraction of *P*-element reads in clusters to the total *P*-element reads, as well as real read numbers (Additional file 2: Figure S2), was higher in Q-KY74 and Q-KY101 than in M′-OM5 and P-Har (Fig. 4b). To compare the characteristics of *P*-harboring piRNA clusters across the lines, we analyzed the number of *P*-element insertions in each piRNA cluster (Fig. 4c). The Q-KY74 genome harbored many *P* elements in unistrand piRNA clusters, but none in dual-strand piRNA clusters. In particular, we found six copies of antisense-oriented *P* elements inserted into the active 100F unistrand cluster (rank 11) (Additional file 2: Figure S2). Q-KY101 carried two copies of antisense-oriented *P* elements in an active dual-strand piRNA cluster, 38C (rank 5), as well as a copy of sense-oriented *P* element in a low activity unistrand piRNA cluster. M′-OM5 carried several copies of both sense- and antisense-oriented *P* elements in active and low activity dual-strand piRNA clusters, but the low number of reads mapped to these suggests that the insertions are polymorphic within the strain (Figs. 4c, Additional file 2: Figure S3). P-Har had a single copy of sense-oriented *P* element in an active dual-strand piRNA cluster and three copies of both sense- and antisense-oriented *P* elements in active and low activity unistrand piRNA clusters, all with low read numbers (Figs. 4c, Additional file 2: Figure S3).

The number of *P*-element copies in piRNA clusters was comparable among the P, M′ and Q strains; however, when read numbers were compared, the fraction of *P*-element reads in clusters to total *P*-element reads was 4- to 5-fold higher in the Q strains than in P and M′ strains (Fig. 4b). These results suggest that the Q strains carry higher occupancies of piRNA-cluster-embedded *P*-element copies within their populations.

### The Q hybrids produced higher levels of *P*-element piRNAs derived from paternal *P* elements

**Fig. 5:**
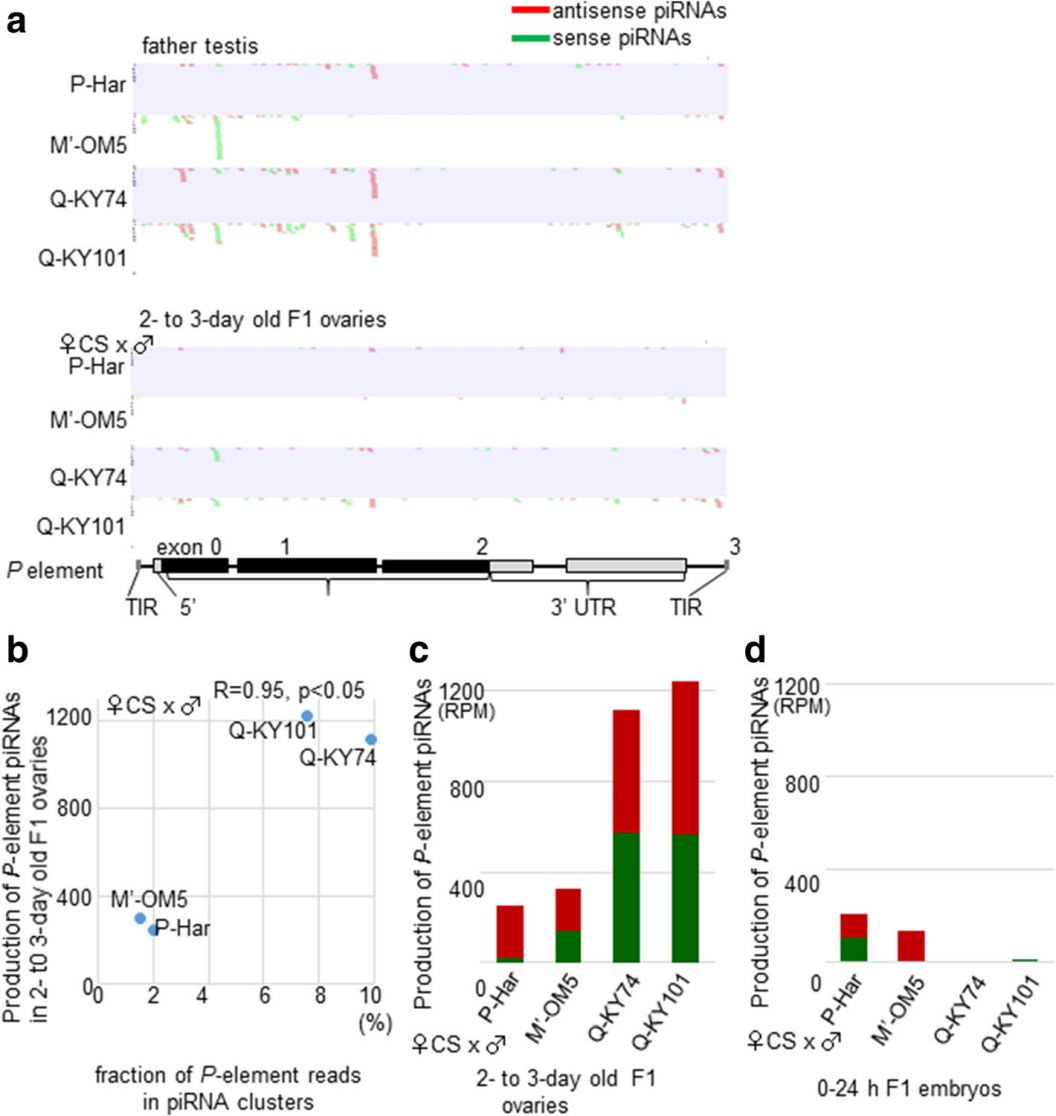
Expression of *P*-element piRNAs derived from *P* elements in F1 hybrids. **a** Small RNA sequencing reads (24–35 nt long) in testes (upper) and F1 ovaries (lower) were mapped to the sense (green) and antisense (red) strands of the *P*-element. The *P*-element structure is schematically shown at the bottom. **b** The positive relationship between occupancy-adjusted relative copy number of piRNA cluster-embedded *P* elements (the fraction of DNA reads supporting *P*-element insertion in clusters, x-axis) and the expression level of *P*-element piRNAs in F1 (CS female x test male) ovaries (y-axis). The Pearson’s R and *p* values are indicated on the top. **c**, **d** The expression levels of *P*-element piRNAs in the ovaries of young (2- to 3 days old) F1 hybrids (**c**) and in 0–24 h whole embryos of F1 hybrid (**d**). *P*-element piRNA reads were normalized by miRNA reads (RPM, reads per million miRNA reads)

To characterize piRNAs produced in F1 hybrids, we deeply sequenced small RNAs in 2- to 3-day-old ovaries from Q, M′, and P F1 hybrids. After removing the miRNAs and fragments of functional RNAs, small RNAs of 24–to 35-nt long were mapped onto the *P* element sequence to identify the *P*-element piRNAs. Because the mother (the M-CS strain) in this cross had no *P*-element in her genome, all *P*-element piRNAs detected in F1 hybrids should have derived from the paternally inherited genome. The analysis revealed the presence of zygotic piRNAs in F1 ovaries of all fly lines (Fig. 5a). The abundances of *P*-element piRNAs differed between the lines, and had a significant positive correlation to the occupancy-adjusted *P* element copy numbers within the piRNA clusters (*R* = 0.95, *p* < 0.05; Pearson’s product–moment correlation test; Fig. 5b). Thus, the two Q hybrids produced > 3-fold more abundant *P*-element piRNAs than the M′ and P hybrids (Fig. 5c). In these Q hybrids, the amounts of sense and antisense piRNAs were similar. For the Q-KY101 hybrids, the very active dual-strand cluster, 38C, likely served as a source of sense and antisense piRNAs. Although the Q-KY74 hybrids harbored nine copies of *P* elements in a unistrand cluster, with eight having an antisense orientation to the cluster transcription, they also produced both sense and antisense piRNAs. It is possible that the *P*-element mRNA was cleaved to serve as sense piRNAs.

Theoretically, it is possible that the detected piRNAs in F1 ovaries were inherited directly from the father’s sperm. To examine this, the small RNAs in testes were also deeply sequenced for the four fly lines. In all lines, testes produced 5- to 10-fold more abundant *P*-element piRNAs than F1 ovaries (Additional file 2: Figure S4); but, in any case, most of the sequences did not closely identify with those in the ovaries of female progeny (Fig. 5a). Therefore, we believe the piRNAs detected in the F1 ovaries were produced mostly de novo, rather than inherited from parental sperm. Consistently observed in Q hybrids, the levels of *P*-element piRNAs were extremely low (< 10 RPM) immediately after fertilization (in 0–24 h whole embryos, Fig. 5d), and then increased to > 1000 RPM (in 2- to 3-day old ovaries, Fig. 5c), most likely by de novo production. Although the degree of increase was less, P and M′ hybrids showed similar trends along the same time line.

In summarizing the results of piRNA analysis, we emphasize that the Q-KY74 and Q-KY101 male parents conferred the ability to produce abundant *P*-element piRNAs in ovaries to their progeny, which well reflects the low GD scores.

### GD scores from both F1 and F2 hybrid progeny were associated with piRNA production in young F1 ovaries

**Fig. 6:**
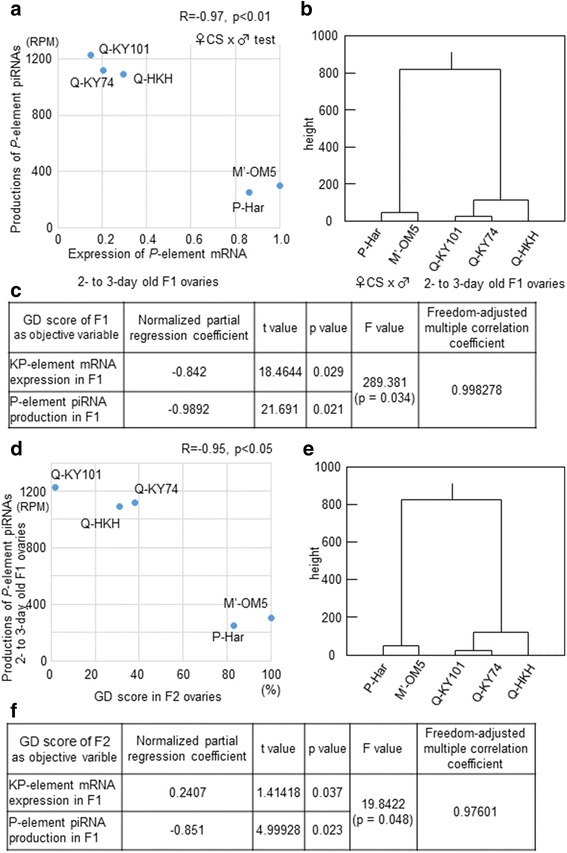
The *KP*-element mRNA and *P*-element piRNA in F1 ovaries are correlated to the GD scores in F1 and F2 hybrids (**a**) The relationship between the expression levels of piRNAs (y-axis) and mRNA (x-axis) of *P* elements in F1 ovaries. The Pearson’s R and p values are shown on the top. **b** A dendrogram of the five natural strains constructed by hierarchical clustering based on the data shown in panel A. **c** The results of the multiple regression analysis for GD scores in F1 (as the objective variable) with the levels of *KP*-element mRNA and *P*-element piRNAs in F1 ovaries (as explanatory objectives). **d** The relationship between the expression levels of piRNAs in F1 hybrids (y-axis) and GD scores of F2 hybrids (x-axis). Pearson’s R and p values are shown on the top. **e** A dendrogram constructed of the five natural strains by hierarchical clustering based on the data shown in panel D. **f** The results of the multiple regression analysis for GD scores in F2 (as the objective variable) with the levels of *KP*-element mRNA and *P*-element piRNAs in F1 ovaries (as explanatory objectives)

To elucidate the relatioship between piRNA production capabilities and GD scores, we first analyzed the relationship between *P*-element mRNA expression and *P*-element piRNA production in 2- to 3-day-old ovaries from F1 hybrids. As shown in Fig. 6a, mRNA expression levels had a significant negative correlation with piRNA expression levels (*R* = − 0.97, *p* = 0.004; Pearson’s product–moment correlation test), suggesting that these piRNAs negatively regulate the levels of *P*-element mRNAs in young hybrid ovaries. Importantly, all three Q hybrids were distinguished from P and M′ strains because they expressed high amounts of piRNAs and very low amounts of mRNAs (Fig. 6b).

These results, together with those from *KP*-element expression analysis (Fig. 3c), suggest that *P*-element piRNAs and *KP* elements are involved in the P-M phenotype. To test this, we performed a multiple regression analysis for GD scores (the objective variable) with the amounts of *P*-element piRNAs and *KP*-element mRNAs from 2- to 3-day-old F1 ovaries (explanatory variables). This revealed that the amounts of both *P*-element piRNAs and *KP*-element mRNAs were effective explanatory variables (for *P*-element piRNAs, partial regression *R* = − 0.99, *t* = 21.7, *p* = 0.02; for *KP*-element mRNA, partial regression *R* = − 0.84, *t* = 18.5, *p* = 0.03; Fig. 6c). Negative R coefficients indicated that the amounts of both *P*-element piRNAs and *KP*-element mRNAs were suppressors of the dysgenesis, and hence repressors of the *P* transposition. Similar t-values suggest that the effectiveness of the *P*-element piRNAs and *KP*-element mRNAs was similar to each other, while the partial R coefficients, at nearly − 1, suggest that high expression of only one of these is sufficient to suppress the dysgenesis. Indeed, for hybrids of low GD scores (M′-OM5, Q-KY101, Q-KY74, and Q-HKH), M′ hybrids showed high *KP* expression in ovaries with low piRNA expression, while Q hybrids showed high piRNA expression with low *KP* expression.

As previously stated (shown in Fig. 1b), GD scores of F2 hybrids also varied between fly strains. Here Q strains showed lower GD scores (2–38%), whereas the P and M′ strains showed 83–100% GD. Thus, using multiple regression we analyzed whether the amounts of the *P*-element piRNAs and *KP*-element mRNAs in F1 ovaries affected the GD phenotype of F2 females (Fig. 6f). Again, the amount of *P*-element piRNA in F1 ovaries was an effective explanatory variable (partial regression *R* = − 0.85, *p* = 0.02) for the F2 phenotype. The amount of *KP* mRNA was also an explanatory variable (*p* = 0.04); however, its effectiveness on the GD phenotype was much weaker (partial regression *R* = 0.24). The t-value for the *KP* mRNA amounts was indeed > 3-fold less than that for piRNA amounts (1.4 vs. 5.0); therefore, the *KP* mRNA in the mothers’ ovaries also affected the GD phenotype of F2 offspring, but the effectiveness was not as pronounced as that observed for the F1 offspring. As a result, we have concluded that *P*-element piRNAs in F1 mother’s ovaries have a large, and possibly major, impact on the GD phenotype of her daughters. Indeed, in a single regression analysis, the piRNA amounts in the F1 mother’s ovaries alone explained well the GD scores of her F2 hybrid offspring (Fig. 6d). We have noted in Fig. 6d and e that the Q strains clustered together.

## Discussion

Although naturally living flies generally carry *P* elements in their genomes, males of the Q and M′ strains in this study demonstrated a higher capacity to suppress the mobilization of introduced *P* elements than the P strain when hybridization occurred with M-strain females. Here, we show that such low *P* inducibility in the three lines of the Q (two lines) and M′ strains was associated with repressive factors derived from the paternal genomes. In particular, we found that the low *P* inducibility in two lines of the Q-strain was strongly associated with a higher level of zygotic piRNAs in young F1 ovaries that contributed to the regulation of *P*-element expression. Interestingly, we found that the level of zygotic piRNAs depended on the *P*-element insertion sites in paternal genomes, and that these piRNAs conferred immunity against *P* transposition in the next generation (F2 hybrids). On the other hand, the low *P* inducibility in one M′-strain line was associated with a higher expression of *KP* elements in F1 ovaries due to a higher copy number of *KP* elements in the paternally inherited genome, where some of *KP* elements are likely inserted into the actively transcribed regions, in addition to the silencing of *FP* elements by harboring them in the transcriptionally inert sites, as previously shown [43]. However, *KP* mRNA levels in the mother’s ovaries did not efficiently protect the daughter’s ovaries from *P*-induced dysgenesis. Srivastav and Kelleher [53] showed that *P* inducibility weakly correlated with the number of *P*-element copies in the genome although the relationship between *P* inducibility and *P*-element insertion sites remains to be explored. In this study, we revealed that, in addition to thesimple copy number, transcriptional activity, and piRNA production ability in the regions surrounding *P*-element copies are important factors.

**Fig. 7:**
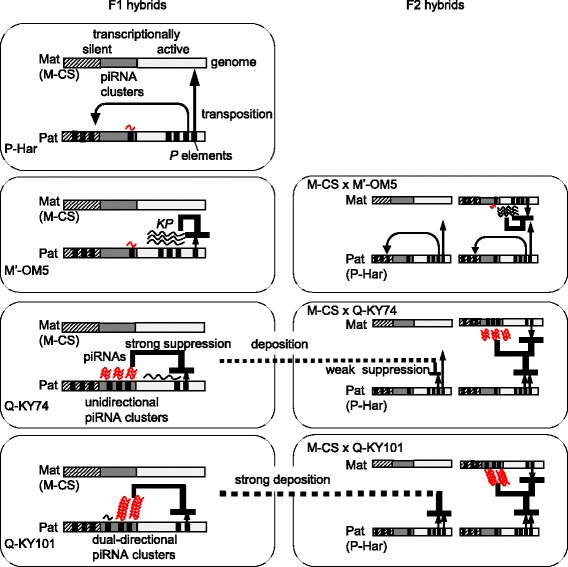
Proposed models for the mechanisms by which the respective paternal genome protects daughters and granddaughters from *P*-element-induced gonadal dysgenesis. Schematic representation of transcriptionally active (light gray box) and silent (oblique-line box) regions, piRNA clusters (dark gray box), and *P* and *KP* elements (thick vertical lines) in the maternally (Mat) and paternally (Pat) inherited genomes, as well as their interactions in F1 and F2 hybrid ovaries. The name of the strain used as the male parent of the F1 progeny are indicated on the left. Short and long wavy lines represent *P*-element piRNAs and *P*-element mRNAs, respectively. Repressive effects are represented by a thick line with an inverted T (inverted T line), where thickness indicates the strength of suppression. Arrows show the transposition of *P* elements. Dotted lines from F1 to F2 hybrids show maternal deposition of piRNAs

Q-KY101 was characterized by low *P*-element expression, and the highest expression of zygotic *P*-element piRNAs in young F1 ovaries; these qualities are associated with high numbers of *P*-element copies harbored in piRNA clusters. In particular, the two *P*-element copies harbored in the 38C piRNA cluster highly active in the germline cells likely account for the highest levels of sense and antisense piRNAs. The offspring of this strain had a very low GD score in F2 ovaries. Assuming that these piRNA-producing *P*-element copies segregate randomly, the F2 phenotype could not be explained solely by the genotype. This suggests that the piRNAs produced in F1 oocytes were deposited to repress the *P*-element expression in F2 progeny (Fig. 7). These results argue that GD of F2 progeny was suppressed by the genomes of their grandfathers carrying *P* elements in their dual-strand (germ-specific) piRNA clusters.

Q-KY74 also is characterized by low *P*-element expression, and high expression of zygotic *P*-element piRNAs in young F1 ovaries, which reflects high *P* element copy numbers harbored in piRNA clusters. While this strain also had a higher number of antisense-oriented *P*-element copies inserted into unistrand piRNA clusters, which are dominant in somatic cells, the Q-KY74 hybrids expressed both-strand zygotic piRNAs in young ovaries. It is possible that, in the young F1 ovaries of Q-KY74 hybrids, many zygotic antisense piRNAs produced from unistrand (soma-specific) clusters induce the production of sense piRNAs by cleaving *P*-element mRNAs. Interestingly, the Q-KY74 strain showed a relatively low (38%) GD score in F2 hybrids. Like Q-KY101, this may be due to piRNA deposition from F1 oocytes (Fig. 7); however, it has been shown that unidirectional piRNA clusters are not active in germ line cells [54]. Previous reports have shown that the piRNAs produced in germline and somatic cells affect each other [55, 56]. Moreover, Malone et al. [4] demonstrated that the low production of antisense piRNAs correlated with the weak deposition of maternal suppressors in F1 progenies, while high both-strand piRNA production correlated with strong deposition of maternal suppressors in F1 progenies. Therefore, there is a possibility that piRNA production in F1 oocytes can be reinforced partially by piRNAs from the unidirectional clusters in ovarian somatic cells, and these oocyte piRNAs are deposited, to some degree, into F2 hybrids (Fig. 7). This means that the difference in the GD scores of F2 hybrids from the Q-KY74 strain and the Q-KY101 strain may stem from a difference in the abundance of the piRNA load in F1 oocytes. Although this presents the possibility of a non-Mendelian inheritance, the moderate GD score of the Q-KY74 strain is explained potentially by Mendelian inheritance as well. Further investigation on the correlation between dysgenic phenotype, piRNA levels, and *P*-element loci of individual F2 hybrids will address this issue.

M′-OM5 hybrids are characterized by low levels of zygotic piRNAs and the active transcription of paternally inherited *P* elements and *KP* elements. Thus, the low levels of piRNAs are not sufficient to decrease the levels of *P*-element mRNA. This is consistent with our previous study on maternal strain effects, where M′-strain females allowed only low levels of piRNAs in F1 hybrids, resulting in *P* susceptibility [38]. However, even with paternally inherited *P* elements considered, the F1 hybrids still presented a low GD score. The genome carried many *KP* elements, some of which resided in transcriptionally active regions, allowing higher *KP*-element expression; therefore, the low *P* inducibility is most likely ascribed to the co-inherited *KP* elements (Fig. 7). However, there is a caveat. In a previous study, all *FP* elements were likely imbedded in transcriptionally silent genomic regions in the M′-OM5 strain, showing low *P* inducibility [43]. Therefore, we propose that, if an active *P*-element is present in the paternally inherited genome, an active *KP* element(s) is required to be co-inherited to suppress the *P*-element activity. Even in such a case, the *KP* elements in the F1 genome would be diluted in F2 hybrids; sufficient amounts of *KP* mRNA are not produced in F2, resulting in GD.

We demonstrated that, in 2- to 3-day-old hybrid ovaries of P-Har, high *P* inducibility was associated with low levels of zygotic *P*-element piRNAs, which is consistent with a previous report by Khurana et al. [27]. Their low piRNA expression is likely because the P-Har genome carries low copy numbers of *P*-elements in piRNA clusters. High percentages of GD in F2 hybrids were affected by this low production of *P*-element piRNAs in F1. It should be noted that the GD score of the P-Har F2 hybrids was 83%, and not 100%, meaning that some progeny had the ability to counteract the *P* transposition. This suggests an involvement of suppressors other than piRNAs, which should be elucidated by further studies.

## Conclusions

Using the *P*-element as a model, our results revealed the importance of zygotically produced piRNAs from the paternal genome to suppress TE activity in *D. melanogaster* progeny. In addition to the well-characterized effects of maternally deposited piRNAs, our results also evoke an interesting possibility that individual TE locations and their insertional polymorphism in natural populations direct the various expressions of piRNAs, leading to variability in the immunizing capacity of their granddaughters against TEs. In nematodes, studies have shown that piRNAs are inherited over many generations [57]. To explore the host-TE battle in natural populations, interesting questions to be addressed include: (1) whether and to what extent the piRNA-producing ability is inherited across generations, (2) whether the transcriptional states of individual TEs are affected by other copies, and if so, (3) whether the altered transcriptional state is inherited, like paramutation [58], as paramutation often involves a class of small RNAs.

## Additional files

Additional file 1: Supplementary methods. (DOCX 21 kb)

Additional file 2: Supplementary figures.  **Figure S1.** The relative abundance of P elements in genomes. P elements present in the respective genome was quantified by qPCR. Their abundance was normalized using the RP49 gene. **Figure S2.** P elements inserted into 100F piRNA cluster in KY74. Closed view the 100F piRNA cluster having 6 copies of P elements in Q-KY74. The nucleotide positions in chr3R are shown on the top. **Figure S3.** P-element reads in piRNA clusters. The read numbers of deep sequencing data that suppot the P-element insertion in the respective piRNA clusters are shown for each fly genome. The clusters are categorized into dual-strand (left) and unistrand piRNA clusters (right). Active piRNA clusters are shown in orange, while low activity piRNA clusters are shown in light blue. The name of piRNA cluster is indicated if appreciable. The rank by piRNA expression level is shown in parenthesis. **Figure S4.** P-element piRNA abundance in testes. The P-element piRNA counts in testes of the respective strains are normalized by miRNA reads. RPM, million mapped miRNA reads. The abundance of sense (green) and antisense (red) piRNAs are colored. (PPTX 133 kb)

## Abbreviations

cross A: CS females x tested males
cross A*: tested females x Har males
M-CS: Canton S
GD: Gonadal dysgenesis
P-Har: Harwich
*P* inducibility: the ability to induce transposition of *P* elements in the germline cells of progeny
M, P, M′, or Q hybrids: the F1 progeny between males of each strain and females of CS
*P* susceptibility: the capacity to allow transposition of *P* elements in the germline cells of progeny
piRNAs: piwi-interacting small RNAs
qRT-PCR: quantitative PCR
RPM: Reads per million miRNA reads
TEs: Transposable elements
