# Supplementary material for: Association of zygotic piRNAs derived from paternal P elements with hybrid dysgenesis in Drosophila melanogaster
Source: Mob DNA. 2018 Feb 6;9:7. doi: 10.1186/s13100-018-0110-y (PMC5800288; doi:10.1186/s13100-018-0110-y)
Supplement: Supplementary file 1 — Supplementary methods. (DOCX 21 kb) [file 13100_2018_110_MOESM1_ESM.docx]

**Supplementary methods**

Wakisaka et al., “Association of zygotic piRNAs derived from paternal *P* elements with hybrid dysgenesis in *Drosophila melanogaster.*”

**PCR and quantitative PCR**

Genomic DNA was extracted from pooled whole fly bodies (20 to 40 flies for each line) using standard methods (Sambrook *et al.* 1989). End-point PCR was conducted with the genomic DNA as a template, the primer sets listed below, and Tks Gflex DNA polymerase (TaKaRa).

<primers for total-*P*-elements>

5ʹ-TCAACGCAGATGCCGTACCT-3ʹ (forward)

5ʹ-CGTCGGCAAGAGACATCCACT-3ʹ (reverse)

<primers for non-KP elements>

5ʹ-TCAACGCAGATGCCGTACCT-3ʹ (forward)

5ʹ-CGACGTTTCGCGCTGCTAAT-3ʹ (reverse)

The PCR products were separated on an agarose gel, and sequenced using Applied Biosystems big dye terminator v3.1 (Applied Biosystems, California, USA) on Applied Biosystems 310 DNA sequencer (Applied Biosystems).

Quantitative PCR was conducted using the genomic DNA and the primers listed below, with SYBR Green for quantification (TOYOBO and Applied Biosystems), on a 7000 HT Fast Real- Time PCR System (Applied Biosystems) CFX96 Real Time System (BIO RAD, California, USA). For each line, three biological replicates of genomic DNA were analyzed, and for each quantification we conducted two technical replicates.

<primers for KP elements>

5ʹ-AACGTGACTGTGCGTTAGGT-3ʹ (forward)

5ʹ-TCAACATCGACGTTTCCACATC-3ʹ (reverse)

<primers for non-KP elements>

5ʹ-GGTGTCTCACGGCGGACTTA-3ʹ (forward)

5ʹ-CGACGTTTCGCGCTGCTAAT-3ʹ (reverse).

<primers for the RP49 gene used for an internal control (Dourlen *et al.* 2012)>

5ʹ-CGGATCGATATGCTAAGCTGT-3ʹ (forward)

5ʹ-GCGCTTGTTCGATCCGTA-3ʹ (reverse)

**Identification of *P*-element insertion sites by deep sequencing**

Genomic regions containing a *P* element and its flanking region were amplified by PCR as described previously (Tsukiyama *et al.* 2013) with minor modifications. The genomic DNA (100 μg) was digested with *Hha*I or *Taq*I (TaKaRa) and ligated to an adaptor duplex DNA (0.25 pmol) with a compatible overhang using 70U of T4 DNA ligase (TaKaRa). The sequences of the adaptor DNAs were as follows:

<*Hha*I adaptor>

5ʹ-CTCGTAGTCGGCACAGGATCACTCCGATACGC-3ʹ

5ʹ-GTGCCGACTACGAGCG-3ʹ

<*Taq*I adaptor>

5ʹ-CGATCGTAGTCGGCACAGCATCACTCCGATACGCTAGCA-3ʹ

5ʹ-ATGCTGTGCCGACTACGAT-3ʹ

Ligation products were purified with AMPure XP (Beckman Coulter, California, USA). The primary PCR was performed using the standard protocol with annealing at 61.0°C, with 10 ng purified DNA, Tsk Gflex DNA polymerase (TaKaRa), the adaptor-specific primer, and the *P*-element-specific primer. Secondary PCR was performed with annealing at 62°C and with 1/10 the volume of primary PCR products, KOD-Plus-Neo DNA polymerase (TOYOBO), another adaptor-specific primer and another *P*-element-specific primer. The primer sequences are as follows:

<for *Hha*I products>

5ʹ-GCGTATCGGAGTGATCCT-3ʹ (adaptor-specific, primary PCR)

5ʹ-CACACTTCGGCACGTGAAT-3ʹ (*P*-element-specific, primary PCR)

5ʹ-GTGATCCTGTGCCGACTAC-3ʹ (adaptor-specific, secondary PCR)

5ʹ-ACAAGCAAACGTGCACTGA-3ʹ (*P*-element-specific, secondary PCR)

<for *Taq*I products>

5ʹ-TGCTAGCGTATCGGAGTGATG-3ʹ (adaptor-specific, primary PCR)

5ʹ-GCTGTCTCACTCAGACTCAA-3ʹ (*P*-element-specific, primary PCR)

5ʹ-ATGCTGTGCCGACTACGAT-3ʹ (adaptor-specific, secondary PCR)

5ʹ-CACTCGCACTTATTGCAAGCAT-3ʹ (*P*-element-specific, secondary PCR)

For deep sequencing, the 300- to 600-bp-long secondary PCR products were purified from an agarose gel using the QIAquick Gel Extraction Kit (QIAGEN), followed by further purification with AMPure XP (Beckman Coulter). Sequencing libraries were prepared from the purified DNA (0.5–1.0 μg) with TruSeq DNA PCR-Free LT Library Prep Kit (Illumina). Pair-end 250-bp sequencing was performed on MiSeq (Illumina).

**RT-PCR and quantitative RT-PCR**

cDNAs were synthesized by superscript III reverse transcriptase (Invitrogen) using total RNA and an oligo-dT primer. These cDNAs were used as the template for duplicate quantitative PCR reactions performed using SYBR Green for quantification (TOYOBO) on a 7000 HT Fast Real-Time PCR System (Applied Biosystems) and CFX96 Real Time PCR Detection System (BIO RAD). The primer sequences are as follows:

<*P* elements>

5ʹ-GTGGGAGTACACAAACAGAGTCCTG-3ʹ (forward)

5ʹ-CGTATCTGCGTGTCCGTGAAGA-3ʹ (reverse)

<*KP* elements>

5ʹ-AACGTGACTGTGCGTTAGGT-3ʹ (forward)

5ʹ-TCAACATCGACGTTTCCACATC-3ʹ (reverse)

<the *RP49* gene as an internal control (Dourlen *et al.* 2012)>

5ʹ-CGGATCGATATGCTAAGCTGT-3ʹ (forward)

5ʹ-GCGCTTGTTCGATCCGTA-3ʹ (reverse).
