# Supplementary material for: Association of zygotic piRNAs derived from paternal P elements with hybrid dysgenesis in Drosophila melanogaster
Source: Mob DNA. 2018 Feb 6;9:7. doi: 10.1186/s13100-018-0110-y (PMC5800288; doi:10.1186/s13100-018-0110-y)
Supplement: Supplementary file 2 — Figure S1. The relative abundance of P elements in genomes. P elements present in the respective genome was quantified by qPCR. Their abundance was normalized using the RP49 gene. Figure S2. P elements inserted into 100F piRNA cluster in KY74. Closed view the 100F piRNA cluster having 6 copies of P elements in Q-KY74. The nucleotide positions in chr3R are shown on the top. Figure S3. P-element reads in piRNA clusters. The read numbers of deep sequencing data that suppot the P-element insertion in the respective piRNA clusters are shown for each fly genome. The clusters are categorized into dual-strand (left) and unistrand piRNA clusters (right). Active piRNA clusters are shown in orange, while low activity piRNA clusters are shown in light blue. The name of piRNA cluster is indicated if appreciable. The rank by piRNA expression level is shown in parenthesis. Figure S4. P-element piRNA abundance in testes. The P-element piRNA counts in testes of the respective strains are normalized by miRNA reads. RPM, million mapped miRNA reads. The abundance of sense (green) and antisense (red) piRNAs are colored. (PPTX 133 kb) [file 13100_2018_110_MOESM2_ESM.pptx]

## Slide 1
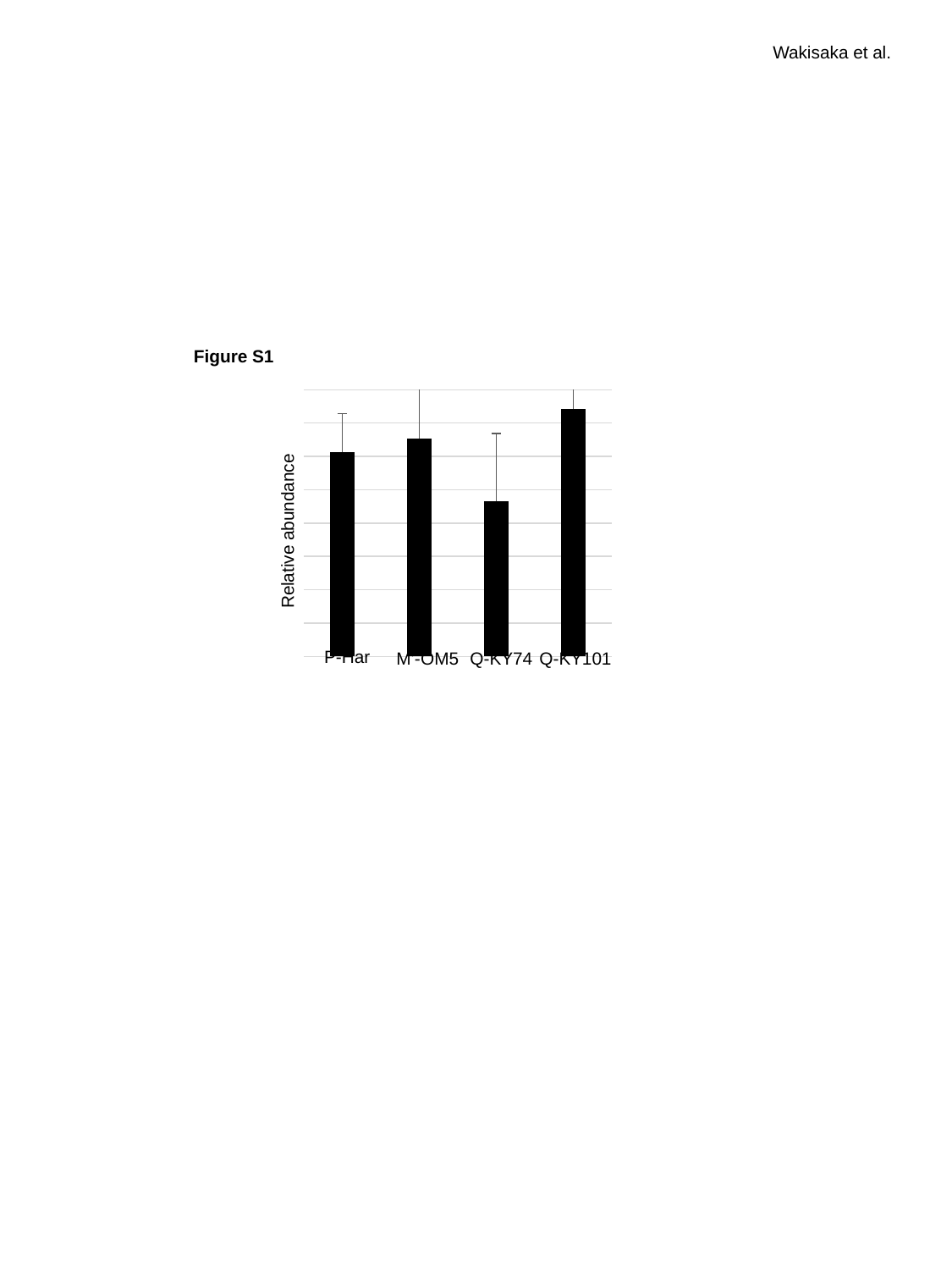

Wakisaka et al.
### Chart
| Category |
|---|Figure S1
### Chart
| Category | av |
|---|---|
| h | 613.262779733372 |
| om | 653.5985035450826 |
| 74 | 465.855847756678 |
| 101 | 743.1443861424663 |Relative abundance
P-Har
M’-OM5
Q-KY74
Q-KY101

## Slide 2
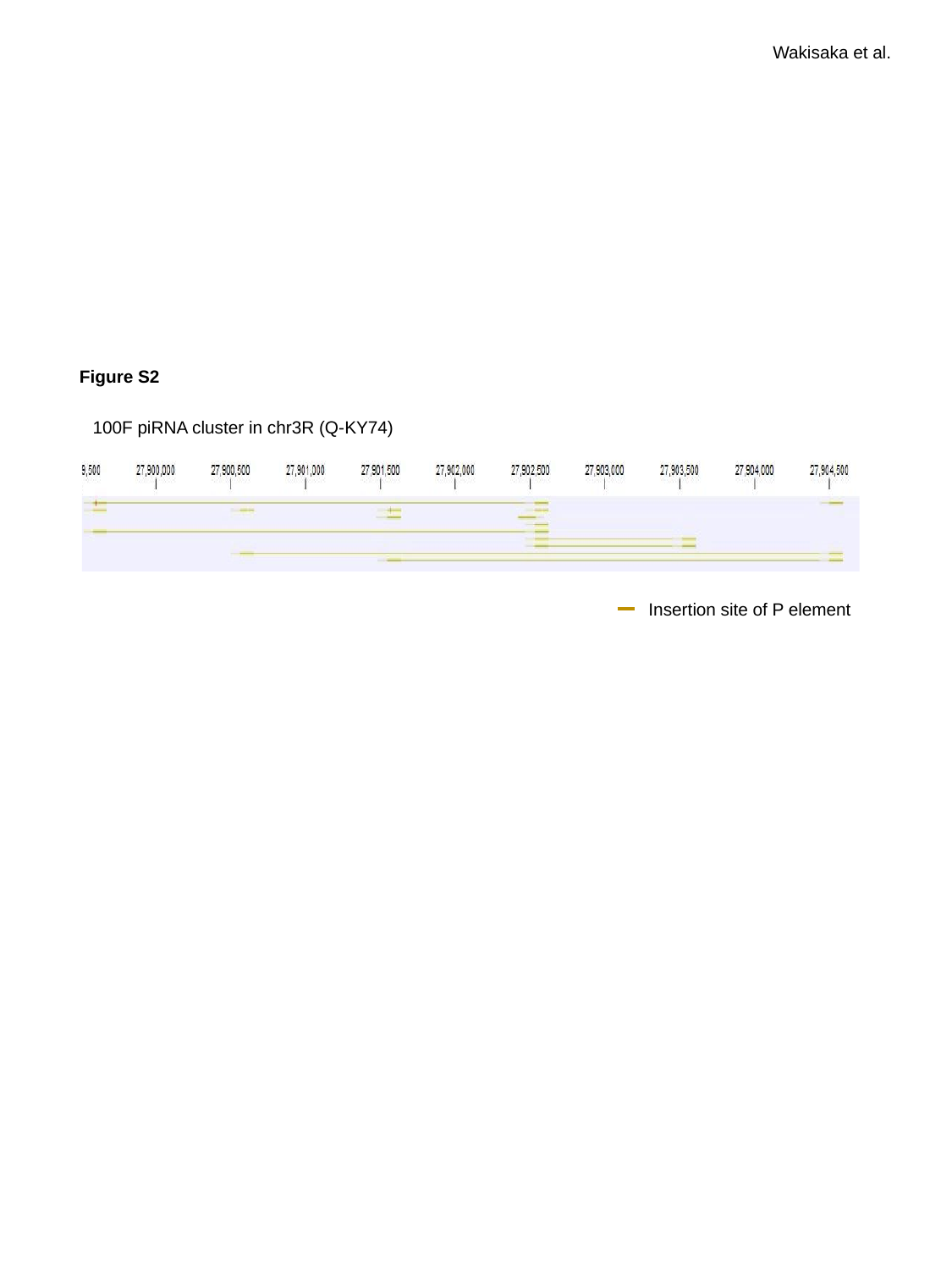

Wakisaka et al.
Figure S2
100F piRNA cluster in chr3R (Q-KY74)
Insertion site of P element

## Slide 3
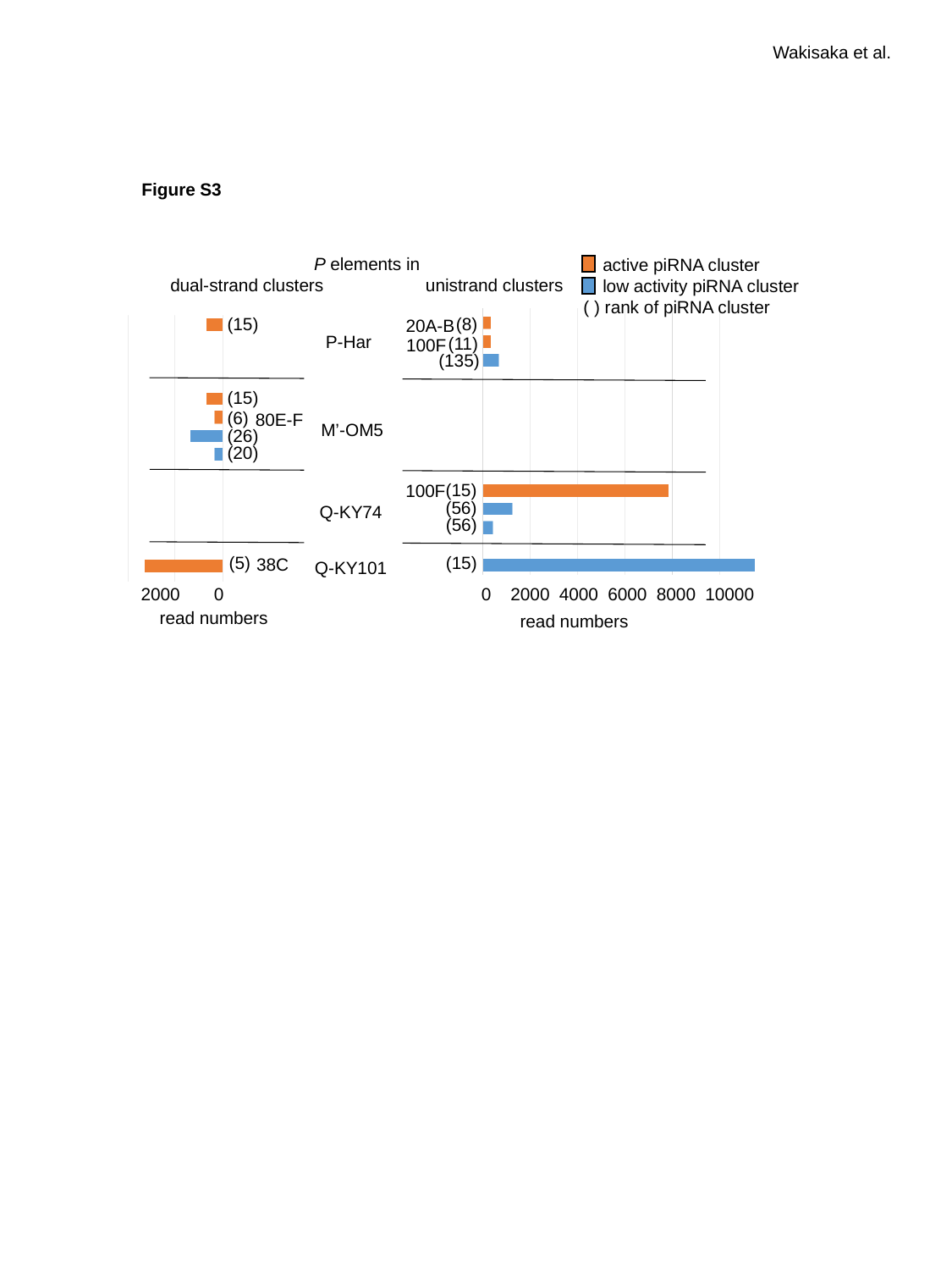

Wakisaka et al.
Figure S3
P elements in
dual-strand clusters unistrand clusters
 active piRNA cluster
 low activity piRNA cluster
( ) rank of piRNA cluster
(15)
(8)
20A-B
P-Har
M’-OM5
Q-KY74
Q-KY101
(11)
100F
(135)
(15)
(6)
80E-F
(26)
(20)
(15)
100F
(56)
(56)
(5)
(15)
38C
2000 0 0 2000 4000 6000 8000 10000
read numbers
read numbers

## Slide 4
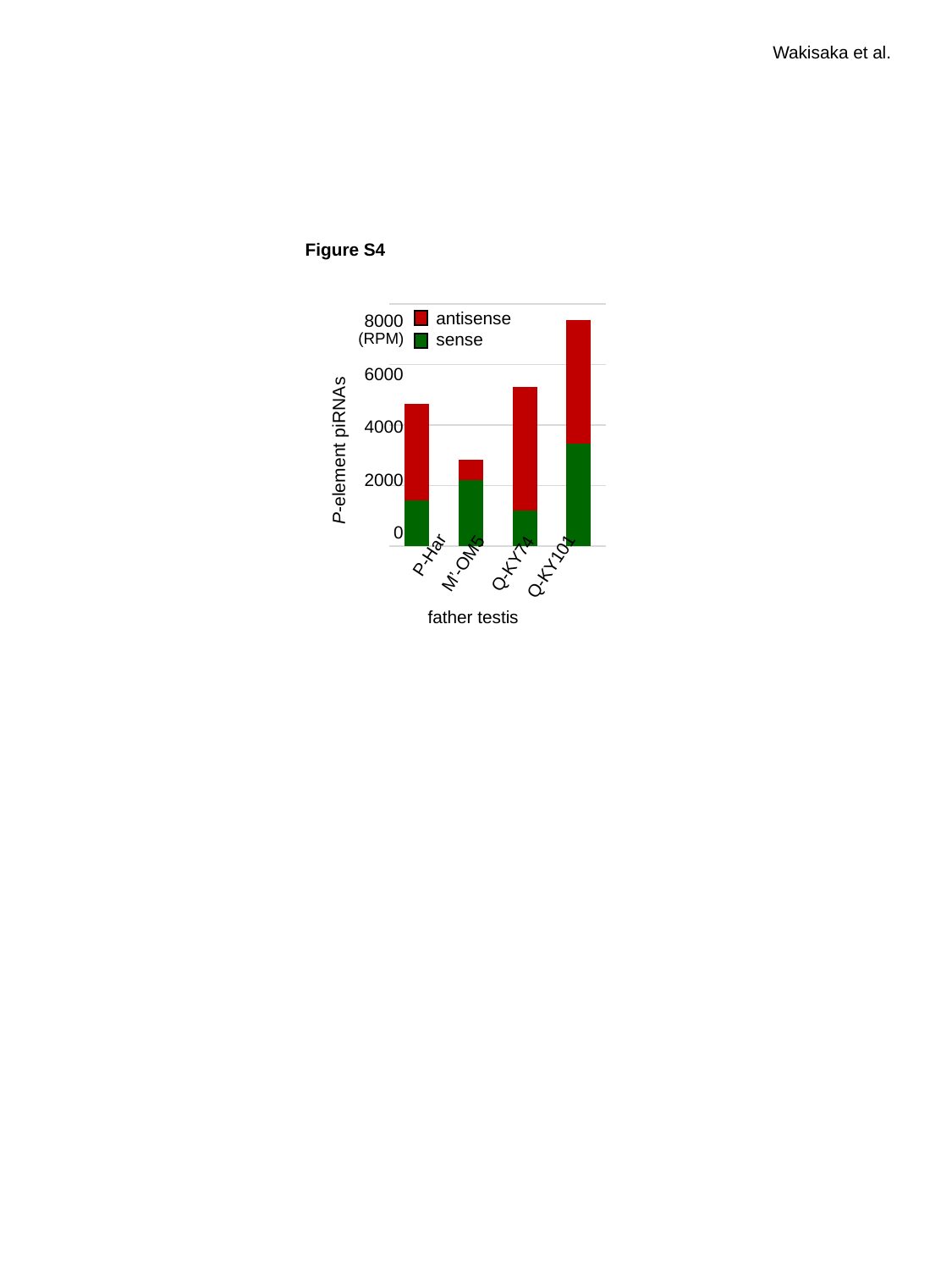

Wakisaka et al.
Figure S4
### Chart
| Category | sense | anti |
|---|---|---|
| h | 1503.156628920734 | 3206.734141697565 |
| om | 2183.67599192452 | 659.2229409583455 |
| ky74 | 1179.453912838356 | 4069.115999292328 |
| ky101 | 3384.26317623053 | 4081.023241925048 |antisense
sense
 8000
 6000
 4000
 2000
 0
(RPM)
P-element piRNAs
P-Har
M’-OM5
Q-KY74
Q-KY101
father testis
